# Assessment of Microbiota Modulation in Poultry to Combat Infectious Diseases

**DOI:** 10.3390/ani11030615

**Published:** 2021-02-26

**Authors:** Laura Montoro-Dasi, Arantxa Villagra, María de Toro, María Teresa Pérez-Gracia, Santiago Vega, Clara Marin

**Affiliations:** 1 Instituto de Ciencia y Tecnología Animal, Universidad Politécnica de Valencia, 46022 Valencia, Spain; laura.montoro@outlook.com; 2 Centro de Calidad Avícola y Alimentación Animal de la Comunidad Valenciana (CECAV), 12539 Castellón, Spain; 3 Centro de Investigación y Tecnología Animal, Instituto Valenciano de Investigaciones Agrarias, 12400 Castellón, Spain; villagra_ara@gva.es; 4 Plataforma de Genómica y Bioinformática, Centro de Investigación Biomédica de La Rioja, 26006 La Rioja, Spain; mthernando@riojasalud.es; 5 Área de Microbiología, Departamento de Farmacia, Instituto de Ciencias Biomédicas, Facultad de Ciencias de la Salud, Universidad Cardenal Herrera-CEU, CEU Universities, Avenida Seminario s/n, 46113 Moncada, Spain; teresa@uchceu.es; 6 Departamento de Producción y Sanidad Animal, Salud Pública Veterinaria y Ciencia y Tecnología de los Alimentos, Instituto de Ciencias Biomédicas, Facultad de Veterinaria, Universidad Cardenal Herrera-CEU, CEU Universities, Avenida Seminario s/n, 46113 Moncada, Spain; svega@uchceu.es

**Keywords:** broiler, growing period, microbiota, farm management, welfare, 16S rRNA analysis

## Abstract

**Simple Summary:**

This research was designed to evaluate the differences in caecal microbiota in broilers reared under two different farm conditions (commercial vs. optimal) during the growing period, using 16 rRNA sequencing analysis. Microbiota composition is affected by stress; for this reason, it could be considered a biomarker of poultry welfare and health. The main results demonstrated that no statistically significant differences were found between groups on microbiota composition from the beginning to the mid-period. However, significant differences were found at the end of growing, when a higher level of microbiota diversity was observed in the optimal farm conditions group. In conclusion, microbiota composition could be an interesting tool to evaluate new management conditions at field level, and could be developed to improve animal welfare during the growing period.

**Abstract:**

Poultry is one of the main agricultural sub-sectors worldwide. However, public concern regarding animal welfare and antimicrobial resistance has risen in recent years. Due to the influence of management practices on microbiota, it might be considered to evaluate poultry welfare and health. Therefore, the objective of this research was to analyse the influence on microbiota balance of broilers under commercial and optimal farm conditions, using 16S rRNA sequencing analysis. The research was performed in two identical poultry houses (commercial vs. optimal). Results showed a higher level of microbiota complexity in the group reared under optimal farm conditions at the end of rearing. Regarding microbiota composition, Firmicutes was the dominant phylum during the entire growing period. However, the second most prevalent phylum was Proteobacteria at the arrival day, and Bacteroidetes from the mid-period onward in both groups. Moreover, the most predominant genera identified were *Oscillospira*, *Ruminococcus*, *Bacteroides*, and *Coprococcus*. In conclusion, it is necessary to optimize farm management as much as possible. Using gut microbiota diversity and composition as biomarkers of animal health could be an important tool for infectious disease control, with the aim of reducing the administration of antibiotics at field level.

## 1. Introduction

Broiler chicken meat is the most consumed meat worldwide, due to the current demand for cheap and safe protein supplies. In fact, in 2020, global poultry meat production increased by 2.6%, and Spain was the fifth largest producer country in the European Union, producing more than one and a half million tons [1,2,3]. These data demonstrate that poultry is the fastest growing agricultural sub-sector. For that reason, producers have historically been driven to intensify farming systems.

However, public concern regarding animal welfare and animal-friendly production systems has increased in recent years [4,5]. Thus, legislation in this area is stricter, and researchers are focused on the study of livestock management conditions to satisfy social concerns and market demands [4,6,7,8]. As defined by the World Organisation for Animal Health, “an animal is in a good state of welfare if it is healthy, comfortable, well nourished, safe, able to express innate behaviour, and if it is not suffering from unpleasant states such as pain, fear and distress” [9]. In this sense, a large number of factors are considered sources of stress in poultry production, such as environmental deterioration, unsuitable social environments, difficulties in accessing essential resources, overcrowding, inadequate temperatures, or diseases [7,10,11].

Historically, to fight against infectious diseases, poultry veterinarians have mainly used antibiotics. However, social demand for antibiotic-free meat has been increasing. For this reason, the main objective is to achieve optimal health and welfare status of the animals in order to increase their resilience. This way, they will be able to cope easily with the environmental risks, including possible pathogens, without antibiotic administration [7,10,12,13,14,15,16].

In this social context, producers are motivated to choose alternative production systems to avoid the drawbacks of more intensive production, while also trying to maintain the profitability of their farms [17,18]. To assess the effect of these alternative management measures, intestinal microbiota composition might be considered as a biomarker of animals’ health and stress status [19,20,21]. It has been demonstrated that any change in the environment directly affects intestinal bacteria balance, and intestinal bacteria balance is known to have an important influence on animal’s health and performance parameters. Thus, the implementation of new and cost effective molecular techniques at field level could help in making rapid and swift management decisions [19,20,21,22,23,24].

Hence, the aim of this study was to analyse the influence on microbiota balance of broilers in standardised commercial farm conditions or under improved farm conditions, using 16S rRNA sequencing analysis.

## 2. Materials and Methods

In this trial, the handling of experimental animals was approved by the Ethical Review Panel of the Directorate-General for Agriculture, Fisheries and Livestock from the Valencian Community by the code 2018/VSC/PEA/0067, according to Spanish Royal Decree 53/2013 [25].

### 2.1. Experiment Design

In this study, two different environmental farm conditions were studied: commercial farm conditions (CFC, house 1:35 kg/m^2^ of final density and non-optimal ventilation parameters, allowing a maximum ammonia concentration of 25 ppm) and optimal farm conditions (OFC, house 2: final density at 17 kg/m^2^ and ventilation within the optimal parameters, allowing a maximum ammonia concentration of 10 ppm).

To this end, a total of 1,062 day-old-chicks (Ross^®^, Aviagen, USA) (males and females) were distributed in two poultry houses in an experimental poultry farm at the Centre for Research and Animal Technology (CITA-IVIA, in its Spanish acronym, Valencian Institute for Agrarian Research, Segorbe, Spain). In each of the houses, 204/531 animals were located in 12 pens with wood shavings as bedding material. The rest of the animals (327/531) were housed in the remaining space also using wood shavings as bedding material to simulate production conditions. According to common practice in poultry production, houses were supplied with programmable electrical lights, automated electric heating, and forced ventilation. The environmental temperature was gradually lowered from 32 °C (1 day) to 19 °C (42 days). Moreover, high biosecurity levels were maintained in the experimental poultry farm during the rearing.

Animals were fed with two different diets according to standard diets for broilers: from hatching day until 21 days post hatch, chicks were fed a pelleted starter diet (Camperbroiler iniciación, Alimentación Animal Nanta, Valencia, Spain), and from 21 days of age to the slaughter day (42 days of age) the poultry were offered a pelleted grower diet (A-32 broiler, Alimentación Animal Nanta, Valencia, Spain). The nutritional composition of the diets is detailed in Table 1. Only one batch of feed per age was provided, no coccidiostats or antimicrobials were added, and all the analyses were assessed before the beginning of the experiment. Feed was supplied ad libitum, but to control feed consumption, it was weighed and added manually. Finally, mortality and the presence of diarrhoea were registered daily, and animals’ weight and feed consumption were recorded at weekly intervals.

### 2.2. Sample Collection and DNA Extraction

To assess the microbiota evolution, animals from each experimental group were sampled at the arrival day (day-old chicks), at the mid-period (21 days old), and at the slaughter day (42 days of age). On arrival day, animals were selected and caecal samples were collected just before being delivered to the houses (30 samples). Samples were then collected again for each treatment (60 samples/group). Ceca were sampled and placed individually in sterile jars.

After sample collection, caecal content was removed and homogenised. Then, pools of six animals from the same experimental group were prepared (5 pools on arrival day and 10 pools/experimental group at the mid-period and at the end of rearing), the DNA of pools content was extracted (QIAamp Power Fecal DNA kit, Werfen, Barcelona, Spain) and frozen at −80 °C for shipment to the Centre for Biomedical Research of La Rioja (CIBIR, in its Spanish acronym, Logroño, Spain), according to manufacturer’s instructions.

### 2.3. 16S rRNA gene Amplification and MiSeq Sequencing

16S rRNA gene amplification and MiSeq sequencing was performed according to Montoro-Dasi et al. [26].

### 2.4. Data Availability

BioProject: PRJNA612272: Assessment of animal husbandry and environmental control as alternatives to antibiotics use in broiler and growing rabbit production. Effect on multi-resistances.

BioSample: SAMN15190317: Commercial and optimal poultry farm conditions. Caecal microbiota characterisation.

## 3. Results

A total of 45 caecal pools were collected, processed, and sequenced: 5 initial samples and 20 per experimental group throughout the growing period. There were no statistical differences between pools from the same experimental group (*p*-value > 0.05). Moreover, the productive parameters obtained were in accordance with the breed standards, and no clinical signs were observed.

### 3.1. 16 rRNA Sequencing

**Figure 1:**
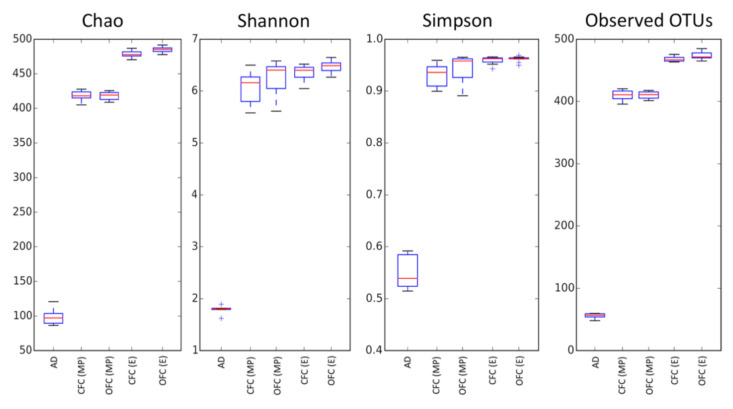
Evaluation of alpha diversity in commercial and optimal farm conditions by using different calculation measures: Chao 1, Shannon, Simpson, Observed Operational Taxonomic Units (OUTs). AD: arrival day; CFC (MP): commercial farm conditions at mid-period; OFC (MP): optimal farm conditions at mid-period; CFC (E): commercial farm conditions at the end of the growing period; OFC (E): optimal farm conditions at the end of the growing period.

The total of sequencing reads of the 45 samples was 21,961,574 (average 274,519.7 reads/sample), with a total of 19,269,620 filtered reads (average 240,870.3 reads/sample), ranging 121,959–477,578 reads. The rarefaction curves were evaluated according to Shannon, Chao1, Observed Operational Taxonomic Units (OTUs), and Simpson biodiversity indexes. Samples from the group 1 (day-old chicks) are at the limit of the rarefaction, leaving a rarefaction number of 54,070 reads (Figure 1).

Rarefaction curves based on the Chao1, Shannon, Simpson, and Observed OTUs biodiversity (Tables S1–S4) showed statistically significant differences (*p*-value < 0.05). The Chao1 alpha diversity index revealed a notable difference between the caecal microbiota diversity depending on the moment of sampling (Table 2).

### 3.2. Variation in Caecal Microbiome Structure between Farm Conditions

Caecal microbiome structures for CFC and OFC at phylum level are represented in Table 3. According to the Kruskal–Wallis and metagenomeSeq tests, no significant differences were found between farm conditions.

At genus level, 58 taxa were identified, and all of them were present in both production conditions. However, we focused on the 25 genera present at an average relative abundance of more than 0.5% in at least one sample group [26,27,28,29,30].

In the total sampling, 5 genera were present only in day-old-chicks, 15 appeared at mid-period, and 7 appeared at the end of the growing period. Moreover, the most common genera identified were *Oscillospira* spp. (8.8%), *Ruminococcus* spp. (4.0%), *Bacteroides* spp. (3.5%), and *Coprococcus* spp. (3.2%).

In day-old-chicks, the most prevalent genera were unclassified members (U.m.) of Proteobacteria phylum (29.4%), U.m. of Firmicutes phylum (13.0%), U.m. of Ruminococcaceae family (6.7%), *Oscillospira* spp. (6.0%), *Clostridium* spp. (5.6%), U.m. of Lachnospiraceae family (5.3%), *Enterococcus* spp. (3.8%), *Ruminococcus* spp. (3.5%) and U.m. of Enterococcaceae family (3.1%).

At mid-period (21 days of age), the predominant bacteria were U.m. of Firmicutes phylum (27.4% and 28.0% for CFC and OFC, respectively), U.m. of Ruminococcaceae family (18.3% and 18.2%), U.m. of Lachnospiraceae family (11.1% and 11.2%), *Oscillospira* spp. (10.4% and 10.3%), *Ruminococcus* spp. (4.4% for both farm conditions), and *Coprococcus* spp. (3.7% for both farm conditions).

Finally, at slaughter day (42 days of age), the most common genera were, likewise, U.m. of Firmicutes phylum (28.0% for both experimental groups), U.m. of Ruminococcaceae family (16.1% for CFC and 15.6% OFC), U.m. of Lachnospiraceae family (9.5% and 9.6%), and *Oscillospira* spp. (8.7% and 8.5%), followed by *Bacteroides* spp. (5.7% and 0.7% for CFC and OFC, respectively), *Ruminococcus* spp. (3.9% for both groups), and *Coprococcus* spp. (3.2% and 3.6%).

**Figure 2:**
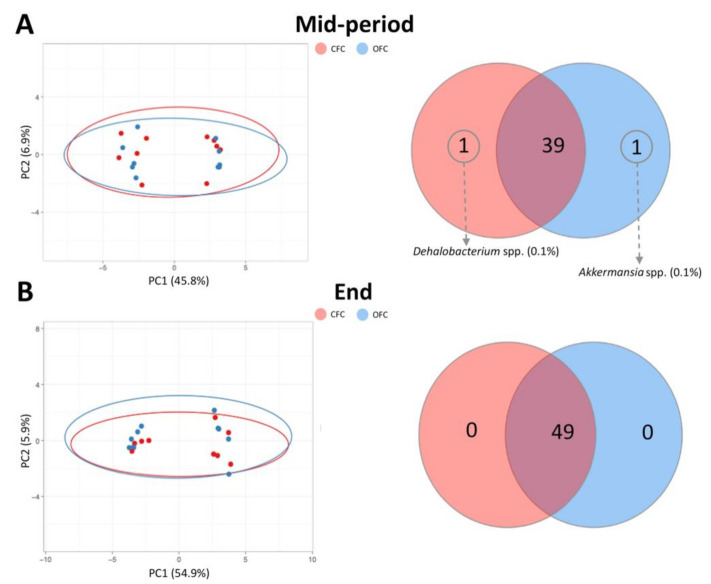
Evaluation of the beta diversity based on Bray–Curtis dissimilarity and comparison of genera presence in commercial and optimal farm conditions. (**A**) Principal Coordinate Analysis graphic and similar vs. different genera for both experimental groups at mid-period. (**B**) Principal Coordinate Analysis graphic and similar vs. different genera for both experimental groups at the end of the growing period.

Finally, to evaluate differences in microbiota between farm conditions, the *R*^2^ values obtained in beta diversity analysis depending on statistical test used were as follows: Bray–Curtis *R*^2^ = 0.84517, Unweighted UniFrac *R*^2^ = 0.79540, and Weighted-UniFrac *R*^2^ = 0.90923 (these data are represented in Figure S1 and detailed in Table S5). Principal Coordinate Analysis (PCoA) of the OTU data for each experimental group reveal different profiles depending on the sampling moment (*p*-value < 0.05). The beta diversity comparisons based on Bray–Curtis dissimilarity and genera presence between both experimental groups throughout the growing period are represented in Figure 2, revealing different profiles depending on the sampling time (*p*-value < 0.05).

## 4. Discussion

The implementation of molecular techniques in microbiology studies allows intestinal bacteria to be evaluated in a “before we saw the tree, now the whole forest” overview. Currently, we are able to observe not only the target bacteria but also all the microorganisms present and their relationship depending on environmental or management conditions.

As described previously, microbiota play a considerable role in animal health. Their composition and richness are directly related with intestinal health, immune system status, and performance parameters. Thus, increasing animal welfare in poultry production above the standards laid down in European Union legislation could improve the intestinal microbiota balance of the animals, thus increasing the resilience of the animals, lessening the prevalence of infectious diseases, and, as a consequence, reducing antibiotic administration in animal production [31,32,33,34,35,36].

Among the different sources of stress, one of the major problems in poultry production is that avian species are particularly sensitive to environmental challenges associated with temperature and stocking density, especially to heat stress. Heat stress is defined as a situation in which temperature and humidity exceed an animal’s comfort zone, and it has a significant effect on the productivity and immunology status of animals, causing multiple physiological disturbances. Heat stress is especially problematic in very humid geographic areas, where achieving optimal ventilation parameters in farms is complicated [24,37,38,39,40].

In this study, animals were reared under two different farm conditions (CFC and OFC) throughout the growing period in order to evaluate the effects of management measures on gut microbiota evolution. There were statistically significant differences in microbiota diversity between farm conditions at slaughter day (42 days of age), when OFC showed a high diversity level. It is well demonstrated that a greater complexity of the gut microbiota is observed as animals grow and that gut microbiota becomes relatively stable as of the mid-period [26,41,42,43,44,45]. However, overcrowding and heat stress present at the end of the growing period usually induce oxidation alteration, which is closely related to intestinal barrier integrity, which is in turn related to gut microbiota [24,46,47,48,49]. Moreover, high stocking density is related to problems in performance and health, possibly caused by poor access to feed and water, abnormal behaviour, and low air and floor quality [11,50,51].

Regarding microbiota composition, the most predominant phyla observed in this research were Firmicutes, followed by Proteobacteria on arrival day and by Bacteroidetes during the rest of the growing period, in line with results reported by other authors [19,20,52,53,54,55]. Moreover, the most predominant genera were also in accordance with the literature [23,24,52,55]. This fact evidences that although the microbiota diversity is low in animals housed according to the European Union legislation, stress levels are not enough to change the microbiota composition.

## 5. Conclusions

In conclusion, microbiota diversity increases throughout the growing period, being relatively stable from the mid-period onwards. However, at the end of the rearing period, a significantly higher level of microbiota complexity was observed in animals reared under optimal farm conditions. Regarding microbiota composition, no statistical differences were observed between experimental groups; for both groups, Firmicutes was the most abundant phylum during the entire research period, Proteobacteria decreased their concentration throughout the growing period, and Bacteroidetes increased. At genus level, the most common groups observed for both management systems were *Oscillospira* spp., *Ruminococcus* spp., *Bacteroides* spp., and *Coprococcus* spp. Thus, it could be recommended that management farm conditions should be reassessed using gut microbiota diversity and composition as biomarkers of animal health. This could be an important tool for infectious disease control, with the aim of reducing the administration of antibiotics at farm level.

## Figures and Tables

**Table 1:** Composition of starter and grower diets.

Analytical Constituents (%)	Diet	
	Starter	Grower
Crude fat	3.5%	3.1%
Crude protein	20.5%	19.4%
Crude fibre	2.6%	3.1%
Crude ash	6.6%	5.0%
Lysine	1.14%	1.13%
Methionine	0.62%	0.51%
Calcium	1.00%	0.78%
Phosphorus	0.69%	0.51%
Sodium	0.15%	0.14%
Metabolic Energy (MJ/kg)	12.20	13.13

**Table 2:** Alpha diversity (Chao 1 index) according to the moment of the growing period in commercial (CFC) and optimal (OFC) farm conditions.

Sampling Moment	Arrival Day	Mid Period	End
CFC	99.6 ^a^	417.5 ^b^	474.8 ^c^
OFC		418.0 ^b^	484.8 ^d^

^a,b,c,d^: different superscripts mean significant differences between groups with a *p*-value < 0.05.

**Table 3:** Taxonomic profiles at phylum level according to sampling moment in commercial (CFC) and optimal (OFC) farm conditions.

Sampling Moment	AD	MP		E		Total
Farm Condition	-	CFC	OFC	CFC	OFC	
*Actinobacteria*	0.0%	0.1%	0.1%	0.2%	0.2%	0.1%
*Bacteroidetes*	3.0%	4.3%	4.1%	9.5%	9.3%	6.1%
*Cyanobacteria*	0.0%	0.0%	0.0%	0.5%	0.5%	0.2%
*Firmicutes*	63.2%	91.5%	91.8%	83.8%	84.0%	83.3%
*Proteobacteria*	33.4%	1.7%	1.7%	2.1%	2.2%	7.9%
*Tenericutes*	0.0%	1.2%	1.2%	1.5%	1.4%	1.1%
*Verrucomicrobia*	0.0%	0.0%	0.1%	0.8%	0.7%	0.3%
Unassigned	0.3%	1.1%	1.0%	1.6%	1.5%	1.1%

AD: arrival day; MP: mid-period; E: end of the growing period. No statistically significant differences were found between farm conditions at phylum level.

## Data Availability

The datasets generated and/or analysed during the current study are available in the NCBI repository, https://submit.ncbi.nlm.nih.gov/subs/sra/SUB7583202/overview.

## Supplementary Materials

The following are available online at https://www.mdpi.com/2076-2615/11/3/615/s1, Figure S1: Evaluation of the beta diversity in commercial and optimal farm conditions. (A) Beta diversity represented by PCoA graphic for both farm conditions at all sampling times. (B) Beta diversity represented by Heatmap for both farm conditions at all sampling times, Table S1: Statistical comparison of alpha diversity between sample groups based on Chao 1 index, Table S2: Statistical comparison of alpha diversity between sample groups based on Shannon index, Table S3: Statistical comparison of alpha diversity between sample groups based on Simpson index, Table S4: Statistical comparison of alpha diversity between sample groups based on Observed OTUs index, Table S5: Statistical comparison between beta-diversity indexes calculated according the different methods.
