# Supplementary material for: Assessment of Microbiota Modulation in Poultry to Combat Infectious Diseases
Source: Animals (Basel). 2021 Feb 26;11(3):615. doi: 10.3390/ani11030615 (PMC7996944; doi:10.3390/ani11030615)
Supplement: Supplementary file 1 [file animals-11-00615-s001.pdf]

animals

MDPI

Supplementary Materials: Assessment of Microbiota Modulation in Poultry to Combat Infectious Diseases

Laura Montoro-Dasi, Arantxa Villagra, María de Toro, María Teresa Pérez-Gracia, Santiago Vega and Clara Marin

Table S1. Statistical comparison of alpha diversity between sample groups based on Chao 1 index.

Group 1	Group 2	Group 1 Mean	Group 1 std	Group 2 Mean	Group 2 std	t stat	*p*-Value
OFC E	AD	484.78	4.27	99.61	12.15	83.56	0.0
CFC MP	AD	417.51	7.43	99.62	12.15	58.25	0.0
CFC E	AD	478.83	4.96	99.62	12.15	79.56	0.0
OFC MP	AD	417.99	5.71	99.62	12.15	64.23	0.0
OFC MP	CFC MP	417.99	5.71	417.51	7.43	0.16	0.88
CFC E	CFC MP	478.84	4.97	417.51	7.43	20.59	3.19×10^{-13}
OFC E	OFC MP	484.78	4.27	417.99	5.71	28.09	2.09E-15
OFC E	CFC MP	484.78	4.27	417.50	7.43	23.55	3.46
CFC E	OFC MP	478.83	4.96	417.99	5.71	24.12	2.49×10^{-14}
CFC E	OFC E	478.83	4.96	484.72	4.27	−2.72	0.02

AD: arrival day; CFC MP: commercial farm conditions at mid period; OFC MP: optimal farm conditions at mid period; CFC E: commercial farm conditions at the end of the growing period; OFC E: optimal farm conditions at the end of the growing period.

Table S2. Statistical comparison of alpha diversity between sample groups based on Shannon index.

Group 1	Group 2	Group 1 Mean	Group 1 std	Group 2 Mean	Group 2 std	t stat	*p*-Value
OFC E	AD	6.48	0.11	1.79	0.09	74.92	0.0
CFC MP	AD	6.07	0.30	1.79	0.09	29.15	4.14
CFC E	AD	6.36	0.14	1.79	0.09	62.06	0.0
OFC MP	AD	6.25	0.32	1.79	0.09	28.13	4.67
OFC MP	CFC MP	6.25	0.32	6.07	0.30	1.23	0.29
CFC E	CFC MP	6.36	0.14	6.07	0.30	2.64	0.03
OFC E	OFC MP	6.48	0.11	6.25	0.32	1.97	0.10
OFC E	CFC MP	6.48	0.11	6.07	0.30	3.81	0.0
CFC E	OFC MP	6.36	0.14	6.25	0.32	0.93	0.42
CFC E	OFC E	6.36	0.14	6.48	0.11	−1.92	0.11

AD: arrival day; CFC MP: commercial farm conditions at mid period; OFC MP: optimal farm conditions at mid period; CFC E: commercial farm conditions at the end of the growing period; OFC E: optimal farm conditions at the end of the growing period.

Table S3. Statistical comparison of alpha diversity between sample groups based on Simpson index.

Group 1	Group 2	Group 1 Mean	Group 1 std	Group 2 Mean	Group 2 std	t stat	*p*-Value
OFC E	AD	0.96	0.01	0.55	0.03	37.28	4.40×10^{-13}
CFC MP	AD	0.93	0.02	0.55	0.03	25.72	1.71×10^{-11}
CFC E	AD	0.96	0.01	0.55	0.03	36.31	3.08×10^{-13}
OFC MP	AD	0.94	0.03	0.55	0.03	23.18	4.84×10^{-11}
OFC MP	CFC MP	0.94	0.03	0.93	0.02	1.06	0.43
CFC E	CFC MP	0.96	0.01	0.93	0.02	3.82	0.0
OFC E	OFC MP	0.96	0.01	0.94	0.03	2.03	0.09
OFC E	CFC MP	0.96	0.01	0.93	0.02	4.27	0.0
CFC E	OFC MP	0.96	0.01	0.94	0.03	1.72	0.16
CFC E	OFC E	0.96	0.01	0.96	0.01	−0.90	0.51

AD: arrival day; CFC MP: commercial farm conditions at mid period; OFC MP: optimal farm conditions at mid period; CFC E: commercial farm conditions at the end of the growing period; OFC E: optimal farm conditions at the end of the growing period.

Table S4. Statistical comparison of alpha diversity between sample groups based on Observed OTUs index.

Group 1	Group 2	Group 1 Mean	Group 1 std	Group 2 Mean	Group 2 std	t stat	*p*-Value
OFC E	AD	473.47	5.85	55.68	4.24	132.33	0.0
CFC MP	AD	409.23	8.54	55.68	4.24	81.33	0.0
CFC E	AD	468.05	4.14	55.68	4.24	167.86	0.0
OFC MP	AD	410.0	5.92	55.68	4.24	111.18	0.0
OFC MP	CFC MP	410.0	5.92	409.23	8.54	0.22	0.84
CFC E	CFC MP	468.05	4.14	409.23	8.54	18.59	1.72×10^{-12}
OFC E	OFC MP	473.47	5.85	410.0	5.92	22.88	6.16×10^{-14}
OFC E	CFC MP	473.47	5.85	409.23	8.54	18.62	1.81×10^{-12}
CFC E	OFC MP	468.05	4.14	410.0	5.92	24.10	2.77×10^{-14}
CFC E	OFC E	468.05	4.14	473.47	5.85	−2.27	0.04

AD: arrival day; CFC MP: commercial farm conditions at mid period; OFC MP: optimal farm conditions at mid period; CFC E: commercial farm conditions at the end of the growing period; OFC E: optimal farm conditions at the end of the growing period.

Table S5. Statistical comparison between beta diversity indexes calculated according the different methods.

Beta-Diversity Matrix	Adonis Test			ANOSIM	
	F-stat	R^2	*p*-Value	Statistic Value	*p*-Value
Bray-Curtis	54.586	0.84517	0.001	0.67777631578947362	0.001
Unweighted-Unifrac	38.876	0.79540	0.001	0.6668026315789474	0.001
Weighted-Unifrac	100.17	0.90923	0.001	0.688736842105263	0.001

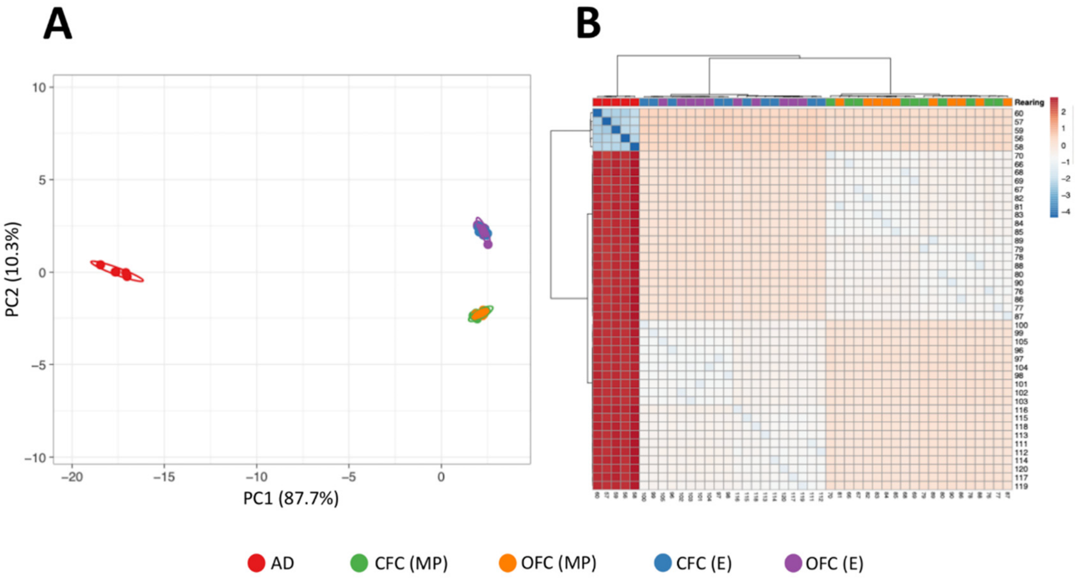

Figure S1. Evaluation of the beta diversity in commercial and optimal farm conditions. (**A**) Beta diversity represented by PCoA graphic for both farm conditions at all sampling times. (**B**) Beta diversity represented by Heatmap for both farm conditions at all sampling times. AD: arrival day; CFC (MP): commercial farm conditions at mid period; OFC (MP): optimal farm conditions at mid period; CFC (E): commercial farm conditions at the end of the growing period; OFC (E): optimal farm conditions at the end of the growing period.
